# Optimize Injection-Molding Process Parameters and Build an Adaptive Process Control System Based on Nozzle Pressure Profile and Clamping Force

**DOI:** 10.3390/polym15030610

**Published:** 2023-01-25

**Authors:** Guan-Yan Liou, Wei-Jie Su, Feng-Jung Cheng, Chen-Hsiang Chang, Ren-Ho Tseng, Sheng-Jye Hwang, Hsin-Shu Peng, Hsiao-Yeh Chu

**Affiliations:** 1 Department of Mechanical Engineering, National Cheng Kung University, Tainan 701401, Taiwan; 2 Department of Mechanical and Computer-Aided Engineering, Feng Chia University, Taichung 407802, Taiwan; 3 Department of Mechanical Engineering, Kun Shan University, Tainan 71070, Taiwan

**Keywords:** injection-molding process, *V/P* switchover point, packing pressure, viscosity index, adaptive process control system

## Abstract

The injection-molding process is a non-linear process, and the product quality and long-term production stability are affected by several factors. To stabilize the product quality effected by these factors, this research establishes a standard process parameter setup procedure and an adaptive process control system based on the data collected by a nozzle pressure sensor and a tie-bar strain gauge to achieve this goal. In this research, process parameters such as the *V/P* switchover point, injection speed, packing pressure, and clamping force are sequentially optimized based on the characteristics of the pressure profile. After the optimization process, this research defines the standard quality characteristics through the optimized process parameters and combines it with the adaptive process control system in order to achieve the purpose of automatic adjustment of the machine and maintain high-quality production. Finally, three different viscosity materials are used to verify the effectiveness of the optimization procedure and the adaptive process control system. With the system, the variation of product weight was reduced to 0.106%, 0.092%, and 0.079%, respectively.

## 1. Introduction

Injection molding is the most popular way for producing thermoplastic polymer products, but due to its non-linear process, many factors will affect the stability of product quality, such as environmental factors, material batches, human factors, process parameters, etc. The injection-molding cycle can be divided into seven steps: mold closing, plasticization, filling, packing, cooling, mold opening, and ejecting. The filling and packing stages have a significant impact on product quality.

Polymer materials have the physical phenomenon of thermal expansion and contraction, and its specific volume will change under different pressures and temperatures. Thus, the final specific volume after cooling will affect product quality, such as product size, product weight, mechanical properties, etc.

The injection-molding parameters can be divided into temperature, pressure, speed, time, and position. According to the *P-V-T* relationship, it is known that the injection-molding parameters are closely related to the product quality. Therefore, the injection-molding parameter setting is essential for the stability of the product weight.

To examine the injection-molding process, installing sensors in the machine or mold is a common approach. Wang [1] et al. defined cavity peak pressure and cavity pressure integral as the quality index and found the significant correlations between cavity peak pressure, cavity pressure integral, and product weight. Chen [2] et al. proposed the peak pressure, viscosity index (which is the pressure–time integral), energy index, and pressure gradient as the quality indexes according to the measurement results of the pressure sensor installed in the nozzle, sprue, and cavity. The results show that the pressure peak is positively associated with product weight. In addition, the viscosity index and energy index are highly correlated with product weight. Chen [3] et al. proposed a method of measuring the clamping force outside the mold and proposed a new method to adjust the timing of the *V/P* switchover point by using a tie-bar elongation profile. Chen [4] et al. mentioned that the clamping force difference value is significantly correlated with product weight. The clamping force difference value obtained by the strain gauge in the tie-bar has the advantage of not needing to invade the injection-molding machine or the mold itself, so it is a way to monitor product weight in real time.

Currently, installing sensors in the machine or mold is a common approach to monitoring the injection-molding process. Based on the measurement results of the sensors, a standard setting procedure for optimizing injection parameters is established to make the product quality more stable. Nian [5] et al. proposed a standard process parameter setup procedure to optimize the injection speed and *V/P* switchover point according to the measurement results of a cavity pressure sensor. The most appropriate injection speed is at the smallest pressure integral, and the appropriate *V/P* switchover point can be determined when the screw position corresponds to the point on the sudden change of the cavity pressure profile. Chen [6] et al. mentioned that the appropriate injection speed caused the slightest pressure difference during the injection stage. For the large L/t ratio (flow length-to-wall thickness ratio) products, determining the proper *V/P* switchover point should be based on the product’s geometry. Huang [7] et al. mentioned that a too-low clamping force setting leads to the product having flashing, high clamping force setting, which leads to venting problems, and a short shot, especially for products of thinner thickness and materials with low viscosity. Chen [8] et al. proposed a regression analysis based on the clamping force difference to determine the clamping force value when processing low-viscosity materials. The method effectively improves the stability of the product weight. Xu [9] et al. mentioned that the product weight is stable when the clamping force shows no peak variation. Huang et al. [10] mentioned that injection speed, *V/P* switchover point, and first-stage packing pressure settings are highly correlated with the cavity pressure profile and product quality. The *V/P* switchover point too early or late will affect the product weight and warpage.

To avoid product instability caused by environmental factors, material batches, and long-term production, it is necessary to introduce the concept of the adaptive process control system. The injection-molding machine can autonomously adjust injection-molding parameters to stabilize product quality. Zhou et al. [11] proposed an adaptive process control system based on the pressure-screw position integral to adjust the packing pressure to stabilize the product weight. Huang et al. [12] proposed an adaptive process control system based on cavity pressure and found that cavity peak pressure, packing, and product weight had high correlation. The system increased the stability of product weight and geometry. Xu et al. [13] established a real-time monitoring and controlling model for viscosity compensation, which can monitor viscosity fluctuations and achieve self-adjustment during the IM process. Su et al. [14] used the finite element analysis software to analyze the tie-bar and defined the clamping force difference as the quality index. The results show that the clamping force difference value is highly correlated with the product weight. Chen et al. [15,16] established the adaptive process control system based on the clamping force difference value and adjusted the *V/P* switchover point and packing pressure to stabilize the product weight. The results show that the clamping force difference value, *V/P* switchover point, and product weight are highly correlated, and adjusting the *V/P* switchover and packing pressure can effectively stabilize the weight of the product. In addition, some research stated that the adaptive process control system is based on the viscosity index according to the nozzle peak pressure measurement results. Chen [17] et al. established the adaptive process control system based on the viscosity index, which is a pressure–time integral. This is highly correlated among *V/P* switchover point, viscosity index, and product weight. Thus, the weight variation can be known through the viscosity index. Tsai et al. [18] established a prediction model by a neural network to predict the quality of the injection-molded product. The prediction model by a neural network is incorporated into the adaptive process control system to stabilize the injection-molded product’s quality. Fan-Jiang et al. [19] proved that applying a single sensor mounted on the nozzle of the injection-molding machine is sufficient to obtain accurate results. By using one sensor on the nozzle of the injection-molding machine, the cost of the adaptive process control system can be reduced, and the product weight variation can be minimized to 0.21%. Su et al. [20] proposed a standard process parameter setup procedure and combined the tie-bar strain gauge and nozzle pressure sensor to reduce the variation of product weight.

The study also refers to some literature on the hardware specifications of the injection-molding machine to construct an adaptive control system. Chen et al. [21] mentioned that using a high sampling rate controller can make the servo-hydraulic system more stable. Lin et al. [22] used a servo-hydraulic system to investigate the performance (response time) of the proposed system in terms of its speed control (filling process), pressure control (packing process), and *V/P* switchover point control (*V/P* transition). The results show that a pull-back distance of 1 mm can be achieved using a sampling frequency of 1000 Hz. Thus, to increase the screw position control, this study utilized the servo valve.

Based on the literature collected above, this study establishes a standard process parameter setup procedure according to the measurement results of a nozzle pressure sensor and a tie-bar strain gauge and based on nozzle peak pressure, timing of peak pressure, pressure difference, and clamping force difference value to optimize the *V/P* switchover point, injection speed, packing pressure, and clamping force. In addition, this study develops an adaptive process control system to minimize the variation of the injection-molding product’s weight. Finally, this research validates the optimization procedure and control system by using three different viscosities’ materials.

## 2. Methodology

### 2.1. P-V-T Relationship

*P-V-T* relationship displays curves composed by pressure, specific volume, and temperature to present its fundamental material property. The specific volume increases when the temperature increases, decreases when the pressure increases, and the specific volume behaves differently as the material passes through the transition temperature. The final specific volume after cooling will affect the product quality, such as product size, product weight, mechanical properties, and so on.

**Figure 1:**
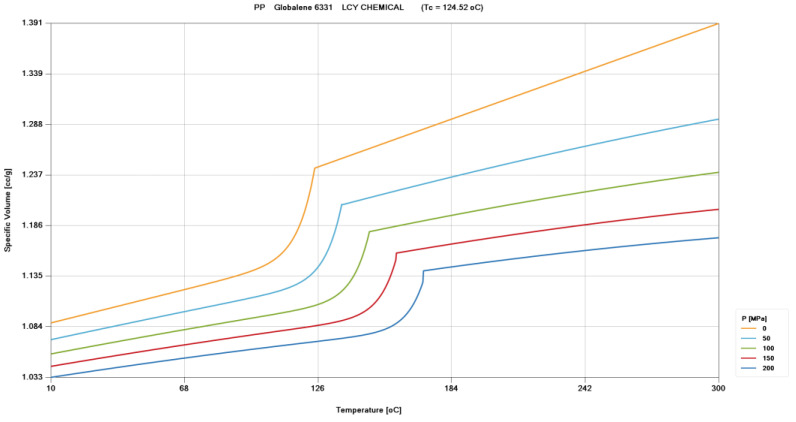
*P-V-T* relationship of PP-6331 (source: Moldex3D 2022).

According to the *P-V-T* relationship, the pressure and temperature will affect the product quality, and many injection-molding parameters are related to them. Therefore, setting the appropriate injection-molding parameters can stabilize the pressure and temperature, and the stability of the product weight will increase. Figure 1 shows the *P-V-T* relationship diagram of polypropylene (Globalene 6331, LCY Chemical Corp., Kaohsiung, Taiwan).

### 2.2. Nozzle Pressure Profile

According to the *P-V-T* relationship, the correlation between pressure and product weight is significant. Therefore, by installing a pressure sensor on the injection-molding machine or the mold, the pressure sensor can monitor the pressure history of the injection-molding process and visualize the product-forming process as much as possible. In recent years, some of the literature has mentioned that installing a pressure sensor at the nozzle end can inspect the product-forming process effectively, and the pressure history of the nozzle end has a significant relationship with the product weight [2,4,17,18,19,20].

#### 2.2.1. Single Nozzle Peak Pressure

**Figure 2:**
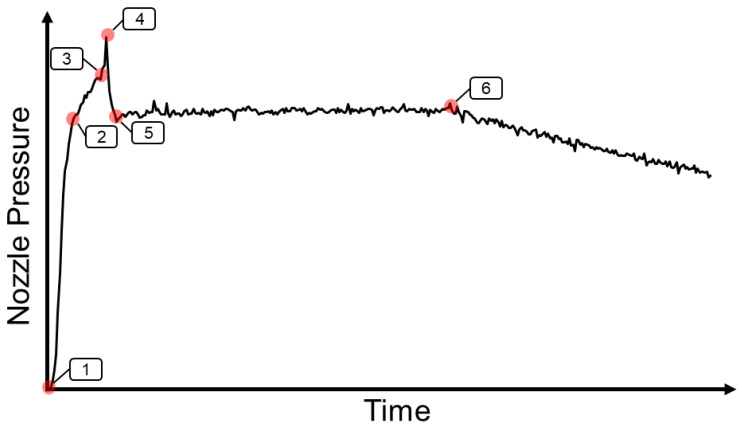
Single nozzle peak pressure profile.

When injection speed was set at 50%, the nozzle pressure profile had only one peak pressure. Figure 2 shows the single nozzle peak pressure profile.

- 1~2: When the screw goes forward to push the melt, the nozzle pressure will increase.
- 2~3: Because the cross-sectional area of the product changes, the melt pressure at the tip of the screw will decrease. In order to avoid the injection speed exceeding the set value, the hydraulic pressure will be restricted, and the nozzle pressure increment will become slow.
- 3~4: When the cavity is completely filled, the behavior of the melt changes from the flowing stage to the compressing stage, which causes a sudden rise in nozzle pressures.
- 4~5: The injection stage is converted to the packing stage.
- 5~6: Packing stage.
- 6: Cooling stage starts.

#### 2.2.2. Double Nozzle Peak Pressure

**Figure 3:**
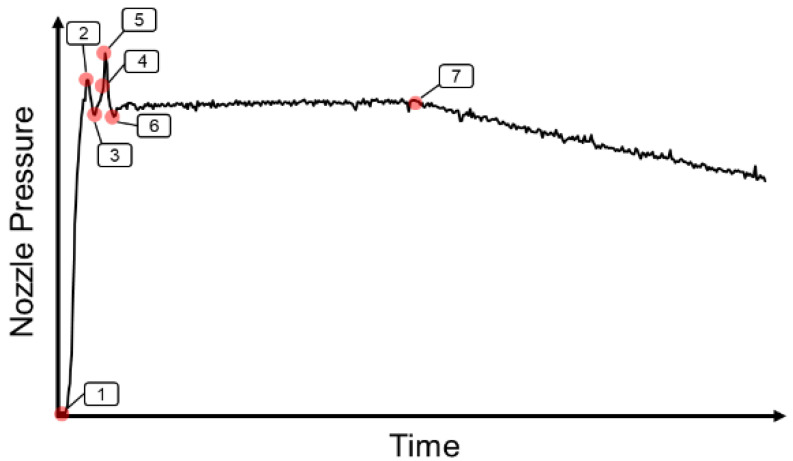
Double nozzle peak pressure profile.

When injection speed was set at 60% or 70%, the nozzle pressure profile had two peak pressures. Figure 3 shows the double nozzle peak pressure profile.

- 1~2: When the screw goes forward to push the melt, the nozzle pressure will increase.
- 2~3: Because the cross-sectional area of the product changes, the melt pressure at the tip of the screw will decrease. In order to maintain the injection speed, the hydraulic pressure will decrease, and the nozzle pressure will decrease.
- 3~4: As the melt enters the cavity, the nozzle pressure will increase.
- 4~5: When the cavity is completely filled, the behavior of the melt changes from the flowing stage to the compressing stage, which causes a sudden rise in nozzle pressures.
- 5~6: The injection stage is converted to the packing stage.
- 6~7: Packing stage.
- 7: Cooling stage starts.

### 2.3. Nozzle Pressure Characteristics

A lot of research uses peak pressure and viscosity index as the characteristics of the pressure profile. The viscosity index is the pressure–time integral, which is used to judge the quality of the injection-molding product online in real time. Schiffers et al. [23] mentioned that the viscosity index is used to observe the variation in the melt flow process during injection stage. Chen et al. [2,4] used the viscosity index to observe the correlation between the product weight under different injection-molding parameters. The results show that the viscosity index and product weight significantly correlate.

**Figure 4:**
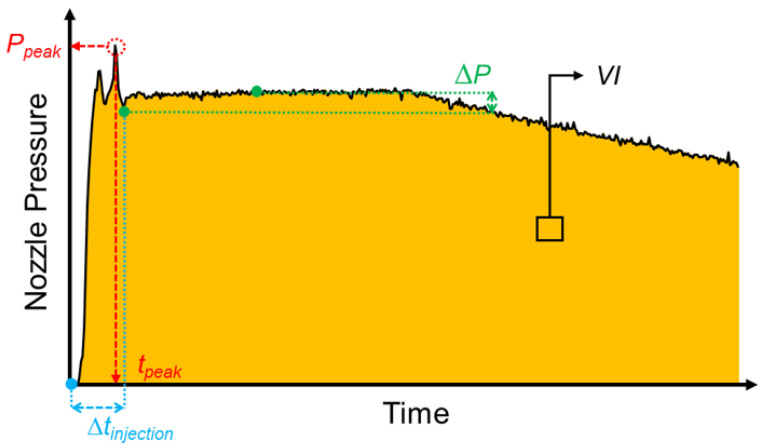
Nozzle pressure profile characteristics.

In order to keep high quality of product, a procedure to define standard process parameters was established based on the results of a nozzle pressure sensor. It is based on quality indexes which are defined as nozzle peak pressure (P*_peak_*), timing of peak pressure (t*_peak_*), pressure difference (∆P), and viscosity index (*VI*) to optimize *V/P* switchover point, injection speed, and packing pressure. In addition, this research develops a control system based on nozzle pressure characteristics that can revise parameters automatically to stabilize product weight. The characteristics are shown in Figure 4.

- Nozzle peak pressure (P*_peak_*): When the cavity is completely filled, the behavior of the melt changes from the flowing stage to the compressing stage, which causes a sudden rise in nozzle pressures. Nozzle peak pressure is used to optimize *V/P* switchover point.
- Timing of peak pressure (t*_peak_*): The time point corresponds to the nozzle peak pressure, and the timing of peak pressure is used to optimize injection speed.
- Pressure difference (∆P): The pressure difference mainly occurs when the injection stage is switched to the packing stage. If different packing pressures are set, the pressure difference response will be different. Pressure difference is used to optimize packing pressure.
- Viscosity index (*VI*): The integral time is from the start of injection to the end of cooling. Viscosity index is mainly used as the quality index of the adaptive process control system to judge the variation of product weight.
  (1) $$\Delta P=P_{packing\_start}-P_{packing\_1.5}$$
  (2) $$VI=\int_{t_{injection\_start}}^{t_{cooling\_end}}P_{nozzle}\left(t\right)dt$$
  where P*_packing_start_* is the pressure when the packing starts, P*_packing_1.5_* is 1.5-s packing pressure, t*_injection_start_* is the time when the injection starts, t*_cooling_end_* is the time when the cooling ends, and P*_nozzle_* is the nozzle pressure.

### 2.4. Clamping Force Difference Value

According to the previous literature, the clamping force will affect the quality of injection-molded products, especially for product’s geometry with materials with low viscosity. The product will generate flashes with too-low clamping force setting, and too-high setting will result in venting problems and short shots [7]. Table 1 shows the status of mold separation. However, the maximum clamping force setting is usually used for business production to lower the time cost, but this setting method may lead to product defects and shortens the life of the mold and the injection-molding machine.

**Figure 5:**
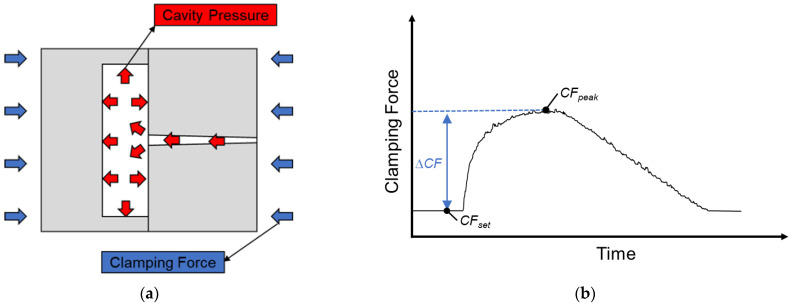
(**a**) Relationship between cavity pressure and clamping force. (**b**) Clamping force difference value [14].

This study installs a strain gauge on the tie-bar in order to obtain the variation of clamping force during the injection-molding process. In addition, the clamping force difference value (Δ*CF*) was defined by the variation of clamping force in order to obtain the appropriate value of clamping force to maintain the quality of product. If the cavity pressure caused mold separation and deformation of the tie-bar, there will be a clamping force peak (*CF_peak_*). The definition of the clamping force difference value is the difference value between the setting value and the peak value of the clamping force (*CF_set_*). The formula and figure of clamping force difference value are shown below and in Figure 5.

(3) $$\Delta CF=CF_{peak}-CF_{set}$$

**Figure 6:**
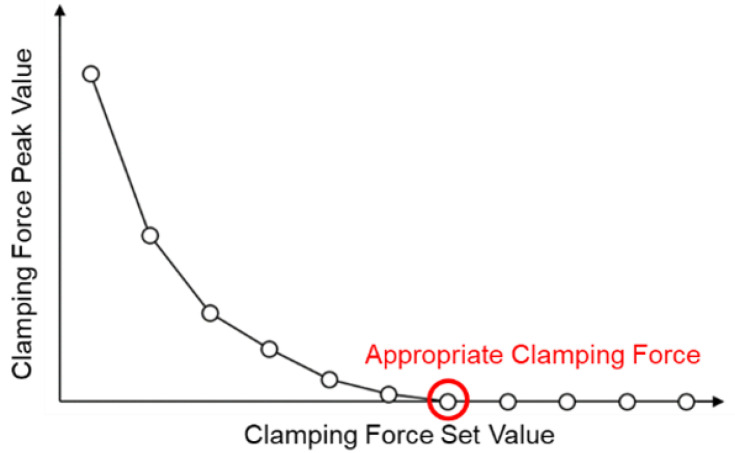
Appropriate clamping force set value [20].

Here, *CF_peak_* is clamping force peak, and *CF_set_* is the setting value of clamping force. The appropriate clamping force defined in this study is when the clamping force difference value is 0, as shown in Figure 6.

## 3. Experiment Setups

### 3.1. Materials

To conduct the experiments, this research used three polypropylenes (PP Globalene 6331-8, 6331, and PT231, LCY Chemical Corp., Kaohsiung, Taiwan) with different viscosities. The melt flow indexes from low to high are 8, 14.5, and 26 (g/10 min).

### 3.2. Equipment

**Figure 7:**
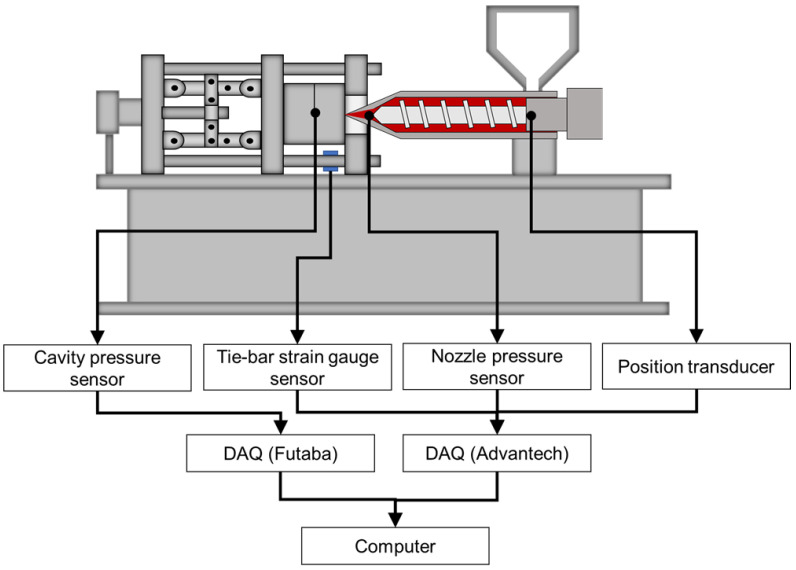
Experiment measurement system.

**Figure 8:**
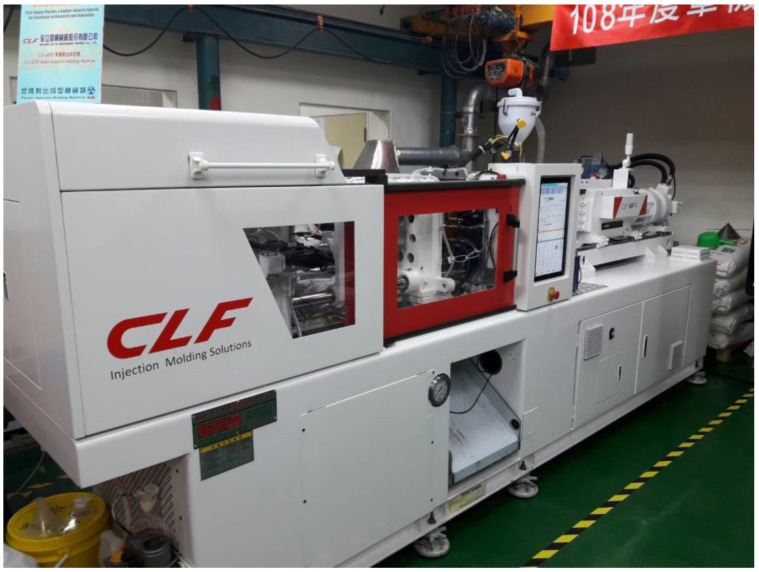
Injection-molding machine.

**Figure 9:**
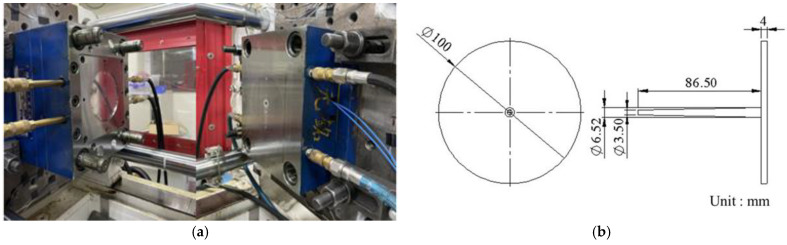
(**a**) Injection mold for the disk sample. (**b**) Dimensions of disk sample.

The experiment measurement system is shown in Figure 7. This research conducts the experiments by using a 60-ton servo-hydraulic injection-molding machine (CLF-60TX, Chuan Lih Fa Co., Ltd., Tainan, Taiwan), as shown in Figure 8. The maximum injection rate was 115 cm^3^/s, the maximum injection pressure was 2951 kg/cm^2^, and the screw diameter was 30 mm. A disk sample was used to conduct the experiment, as shown in Figure 9. To obtain the pressure of melt, this research installs a pressure sensor (PT4656XL, Dynisco, USA) on the nozzle. To obtain the strain, a strain gauge (GE1029, Gefran, Italy) was installed on the tie-bar. A pressure sensor (SSB01KN08X06, Futaba, Japan) was mounted on the cavity to measure the melt pressure. To obtain the pressure from the nozzle and data of the clamping force, a DAQ (USB-4716, Advantech Co., Ltd.) was used. A data acquisition module (MPS08, Futaba, Japan) was used to obtain the cavity pressure sensor data.

## 4. Experiment Results and Discussion

The traditional practice of an engineer to start an injection-molding job begins with machine preheating, parameter setting, and machine calibration, etc. It takes a lot of time and material to find the best process parameter setting before production, and the setup process needs to rely on the experience of engineers. In this study, the procedure was optimized by establishing a standard process parameter setup procedure through the use of process signals from the sensors installed in the machine, which can find the proper process parameters in a short period of time. This procedure does not require experienced engineers, but anyone with basic concepts can operate. This procedure not only saves labor and material costs but also shortens the time required to start production.

In this research, the standard process parameter setup procedure is mainly arranged according to the injection process. The first process parameter optimized is the *V*/*P* switchover point, which mainly affects the material quantity in the injection stage. The second optimized process parameter is the injection speed, which determines whether the melt can completely fill the mold cavity at the injection stage. The third optimized process parameter is the packing pressure, which is mainly used to avoid shrinkage or overpacking of the parts. After the optimization of the above parameters, a set of close-to-optimal process parameters can be obtained. After finding the close-to-optimal process parameters, clamping force needs to be properly set up to avoid mold separation during production.

### 4.1. V/P Switchover Point Optimization Experiments

During the injection-molding process, it is important to decide the appropriate *V/P* switchover point to stabilize the product weight. The theoretical *V/P* switchover point is at 98% of the cavity volume. The product will have flash or shrinkage if the *V/P* switchover point setting is too late or too early. This research determined the appropriate *V/P* switchover point under different *V/P* switchover points setting and 50% injection speed setting. The processing parameters are shown in Table 2.

**Figure 10:**
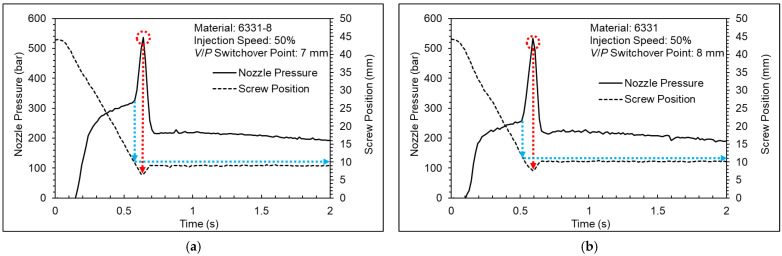

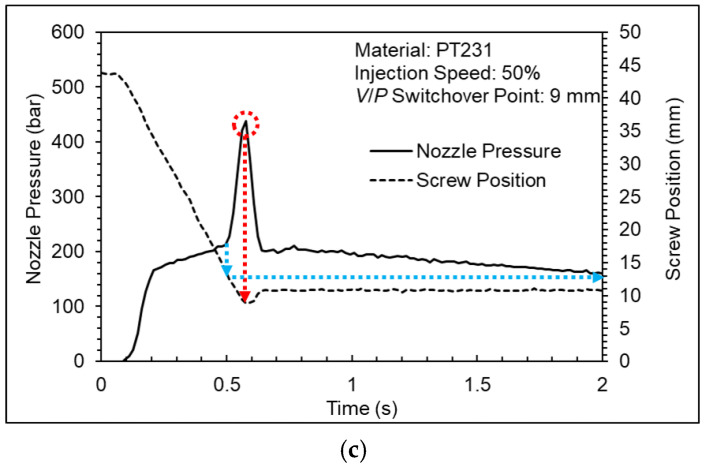
Nozzle pressure profile and screw position profile at the overfilling *V/P* switchover point ((**a**) 6331−8, (**b**) 6331, and (**c**) PT231).

Figure 10 shows a nozzle pressure profile and screw position profile at the overfilling *V/P* switchover point. The experiment results show that when the cavity is completely filled, the behavior of the melt changes from the flowing stage to the compressing stage, which causes a sudden rise in nozzle pressures. The appropriate *V/P* switchover point can be determined when the screw position corresponds to the point on the sudden change of the pressure profile.

**Figure 11:**
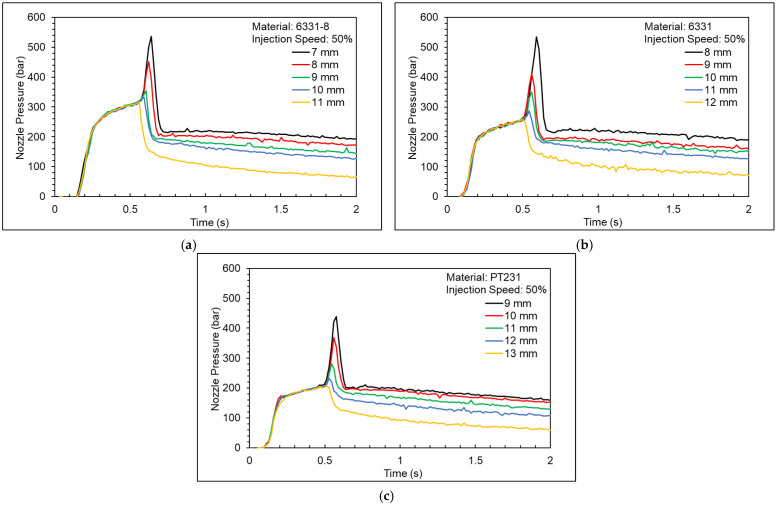
Nozzle pressure profile at different *V/P* switchover points ((**a**) 6331−8, (**b**) 6331, and (**c**) PT231).

**Figure 12:**
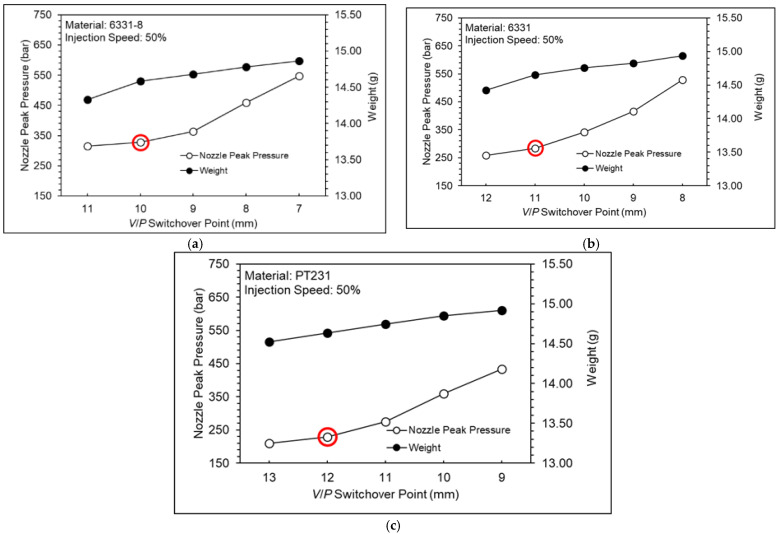
Nozzle peak pressure and product weight at different *V/P* switchover points ((**a**) 6331−8, (**b**) 6331, and (**c**) PT231).

Figure 11 shows a nozzle pressure profile at different *V/P* switchover points. Figure 12 shows a nozzle peak pressure and product weight at different *V/P* switchover points. If the nozzle peak pressure is too high, the cavity will be overfilled, and it will make some flash on the product. In addition, if there is no peak pressure in the nozzle pressure profile, the cavity will be underfilled and create denting of the product. Therefore, the appropriate *V/P* switchover point is defined as when the nozzle pressure has a small peak value. For materials 6331-8, 6331, and PT231, the appropriate *V/P* switchover points are 10 mm, 11 mm, and 12 mm, respectively.

### 4.2. Injection Speed Optimization Experiments

Injection speed is a main indicator in the production cycle of injection molding. Increasing injection speed can shorten the production cycle time of injection molding. However, too fast of an injection speed may lead to over-compression of the melt. In this study, materials with different viscosities and different injection speeds are used to observe the variation of the nozzle pressure profile in order to find the optimum injection speed. The experiment parameters are shown in Table 3. The *V/P* switchover point setting is based on the previous experiment.

**Figure 13:**
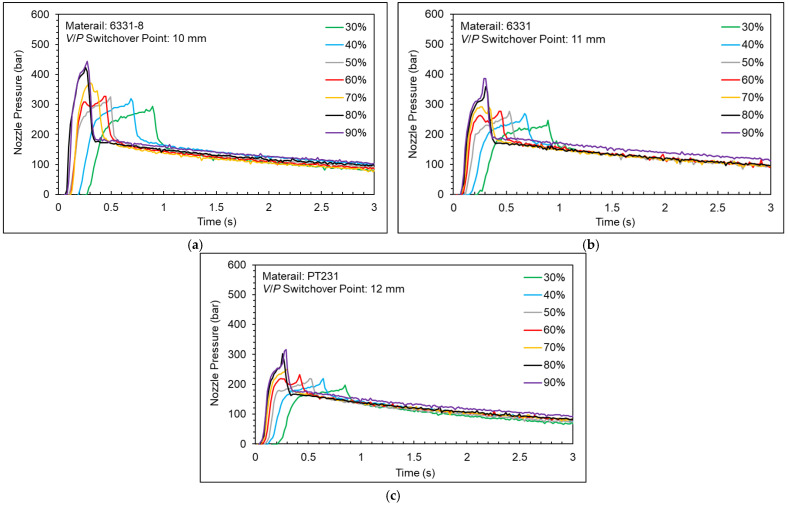
Nozzle pressure profile at different injection speeds ((**a**) 6331−8, (**b**) 6331, and (**c**) PT231).

**Figure 14:**
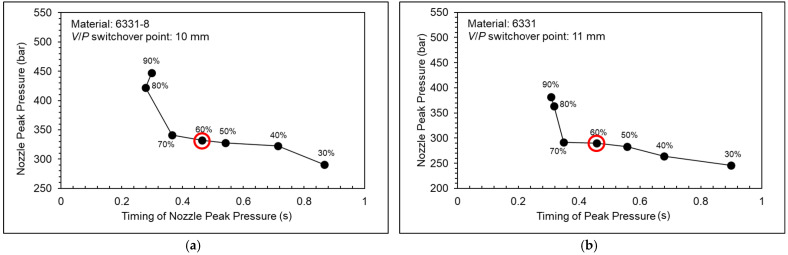

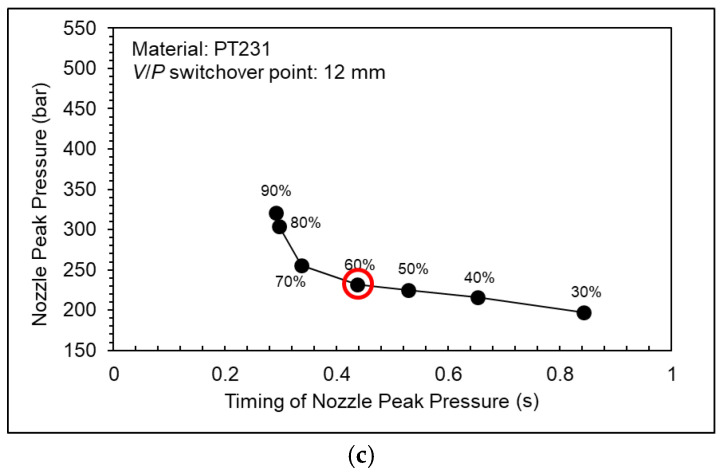
Nozzle peak pressure and timing of nozzle peak pressure at different injection speeds ((**a**) 6331−8, (**b**) 6331, and (**c**) PT231).

Figure 13 shows a nozzle pressure profile at different injection speeds. Figure 14 shows a nozzle peak pressure and timing of nozzle peak pressure at different injection speeds. As shown in the experiment results, the nozzle peak pressure will increase slightly, and the timing of peak will decrease significantly while the injection speed increases under the condition of the injection speed being under 70%. The nozzle peak pressure will increase significantly, and the timing of peak will decrease slightly while the injection speed increases under the condition of the injection speed being above 70%. Under the high injection speed setting, the nozzle peak pressure will rise sharply, and the cavity will be overfilled because of the inertia of the screw and the melt.

To prevent the over-compression of melt and to decrease the injection cycle time, the injection speed needs to be adjusted. Therefore, for the 6331-8, 6331, and PT231 materials, the appropriate injection speed is 60%.

### 4.3. Packing Pressure Optimization Experiments

The packing stage is after the filling stage and the *V/P* switchover point. During this stage, the product will shrink when the melt gradually cools during the injection-molding process. To avoid shrinkage of the product, the injection-molding machine will compensate the melt into the cavity with a certain pressure until the gate is solidified.

If the packing pressure setting is too large, the product will have flashes. If the packing pressure setting is too low, the product will have shrinkage. The packing pressure experiment parameters are shown in Table 4. The setting of the *V/P* switchover point and injection speed is based on previous experiments.

**Figure 15:**
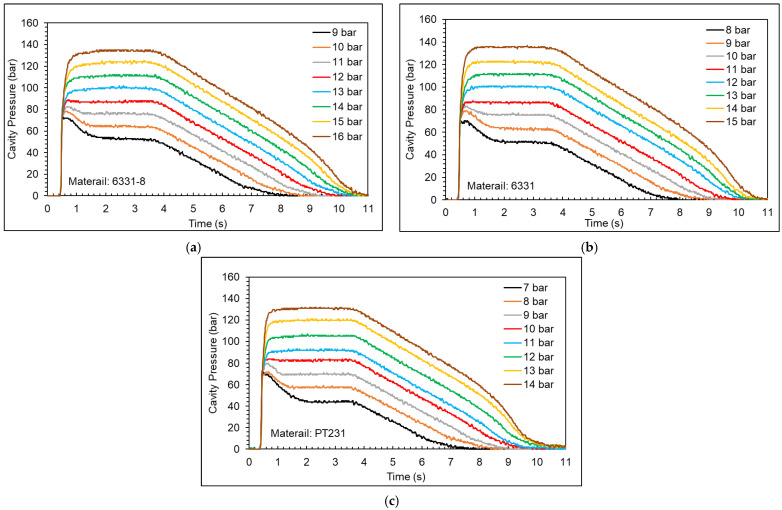
Cavity pressure profiles at different packing pressures ((**a**) 6331−8, (**b**) 6331, and (**c**) PT231).

Figure 15 shows the cavity pressure profiles (full results are shown in the Appendix A). Taking the 6331 material as an example, when the packing pressure is set to 8 bar, 9 bar, and 10 bar, the cavity pressure profile will have a peak because the packing pressure is not high enough to make the melt flow into the cavity, causing the product to shrink. When the packing pressure is set to 11 bar, the cavity pressure profile will gradually rise and then remain flat. There will be no obvious shrinkage in the appearance of the product, which means that the packing pressure setting is enough to compensate for the melt flow into the cavity. 

**Figure 16:**
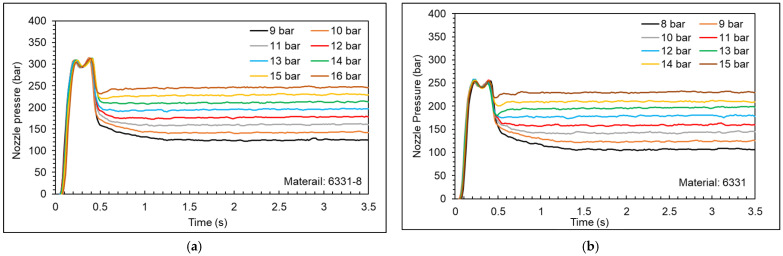

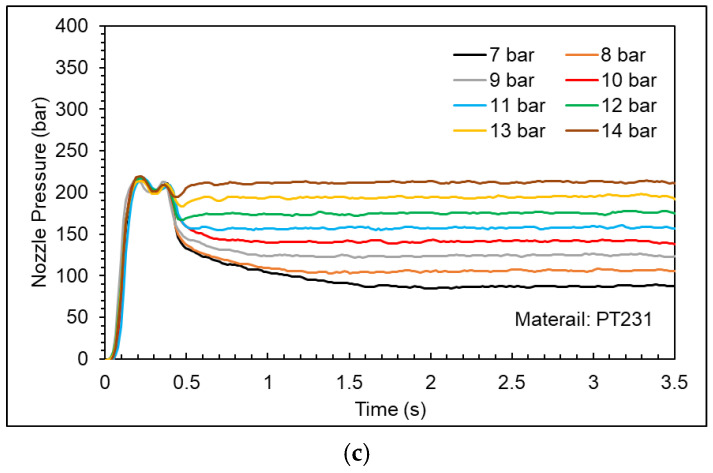
Nozzle pressure profile at different packing pressures ((**a**) 6331−8, (**b**) 6331, and (**c**) PT231).

**Figure 17:**
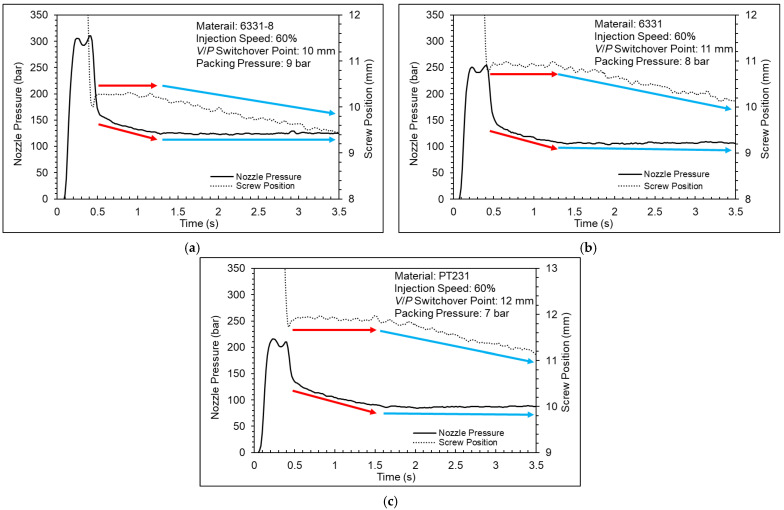
Nozzle pressure profile and screw stroke profile ((**a**) 6331−8, (**b**) 6331, and (**c**) PT231).

**Figure 18:**
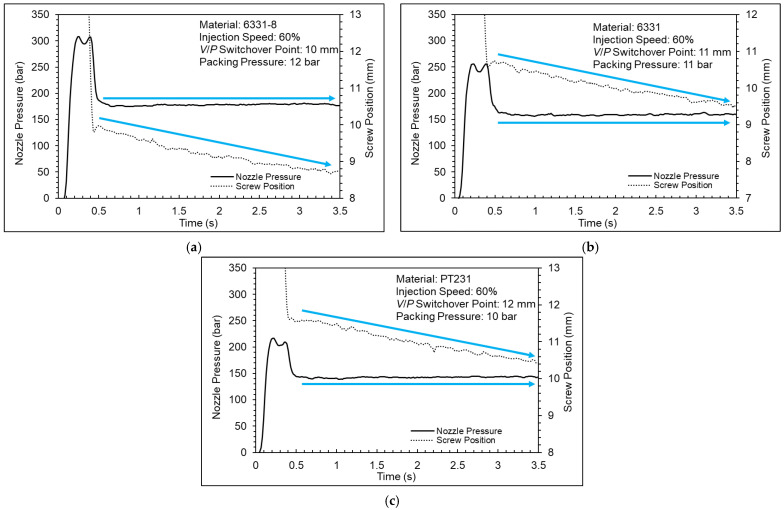
Nozzle pressure profile and screw stroke profile at the sufficient packing pressure ((**a**) 6331−8, (**b**) 6331, and (**c**) PT231).

**Figure 19:**
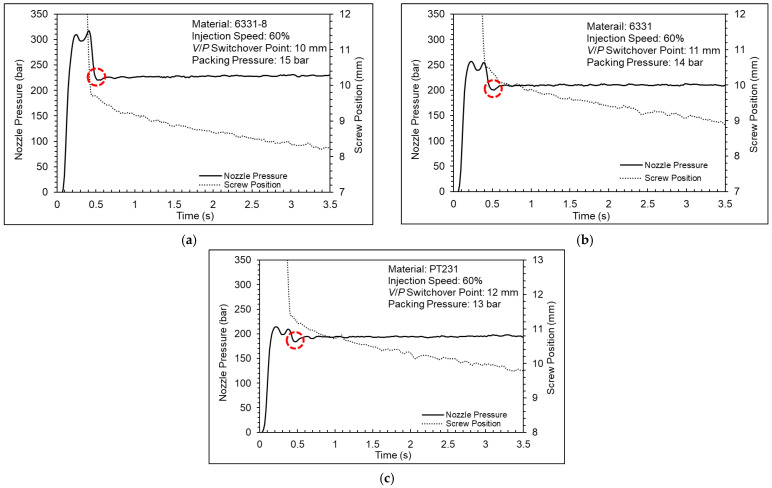
Nozzle pressure profile and screw stroke profile ((**a**) 6331−8, (**b**) 6331, and (**c**) PT231).

Figure 16 shows the nozzle pressure profiles at different packing pressures (the full results are shown in the Appendix A). Taking the 6331 material as an example, when the packing pressure is set at 8 bar, 9 bar, and 10 bar, it takes a period of time for the nozzle pressure to stabilize. In order to understand the reason for this phenomenon, the nozzle pressure profile is compared with the screw position profile (shown in Figure 17, Figure 18 and Figure 19).

Figure 17 shows a nozzle pressure profile and screw position profile. The results show that the nozzle pressure profile and screw position profile can be divided into two parts during the packing stage. When the nozzle pressure continues to reduce, the screw position remains solid; when the nozzle pressure stabilizes, the screw position will move forward. The reason is the insufficient setting of the packing pressure. The pressure of the cavity was greater than the pressure of the barrel at the beginning; therefore, the melt could not be compensated into the cavity nicely by screw. However, if the product gradually cools and shrinks, the screw will be able to move forward. Figure 18 shows a nozzle pressure profile and screw position profile. The results can be observed that the screw can go forward at the beginning and the melt could be compensated into the cavity nicely when the packing pressure is sufficient. Figure 19 shows a nozzle pressure profile and screw position profile. The results show that the nozzle pressure profile will drop and then rise between the injection stage and the packing stage. The reason may be due to the response generated by the PID control of the injection-molding machine.

**Figure 20:**
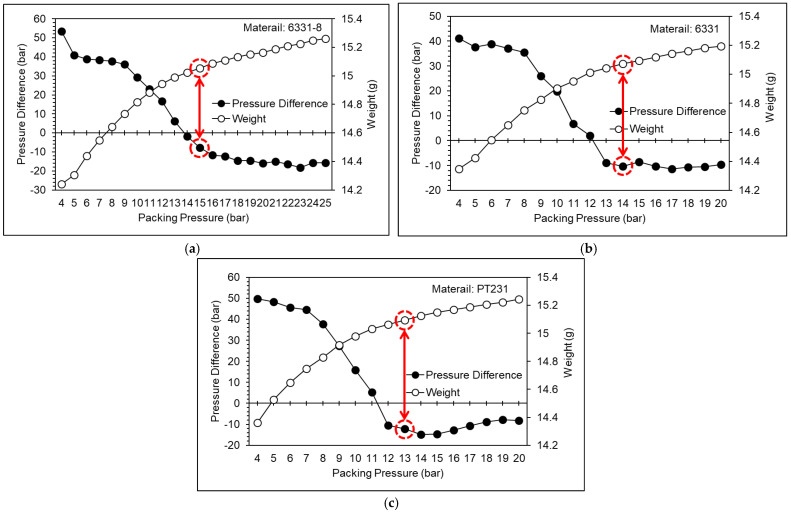
Pressure difference and product weight at different pressures ((**a**) 6331−8, (**b**) 6331, and (**c**) PT231).

According to the results of the cavity pressure profile and the nozzle pressure profile, the appropriate packing pressures for the 6331-8, 6331, and PT231 materials are 12 bar, 11 bar, and 10 bar, respectively. However, when the packing pressure is increased, the product weight will still increase significantly. This means the cavity is still not saturated. Therefore, the packing pressure setting should also consider the product quality. Figure 20 shows a pressure difference and product weight at different pressures. According to the results of the pressure difference and product weight, the variation of the product weight is significantly converged, and the appropriate packing pressures for the 6331-8, 6331, and PT231 materials are 15 bar, 14 bar, and 13 bar, respectively.

### 4.4. Clamping Force Optimization Experiments

Clamping force will affect the quality of injection-molded products, especially for products with geometry with thinner thickness and materials with low viscosity. Too-low clamping force setting leads to the product having flashes, and high clamping force setting leads to venting problems and short shots. The clamping force experiment parameters are shown in Table 5. The setting of the *V/P* switchover point, injection speed, and packing pressure is based on previous experiments.

**Figure 21:**
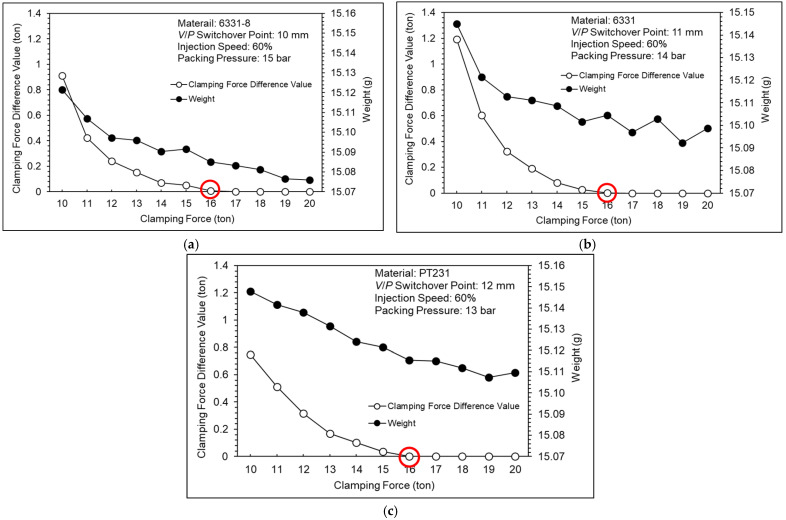
Clamping force difference value and product weight at different clamping forces ((**a**) 6331−8, (**b**) 6331, and (**c**) PT231).

Figure 21 shows a clamping force difference value and product weight at different clamping forces. As shown in the experiment results, the clamping force difference value will decrease, and the product weight will also decrease while the clamping force increases. In addition, the clamping force difference value has approached zero at a clamping force of 16 tons. This means that the clamping force is enough during the injection-molding process.

**Figure 22:**
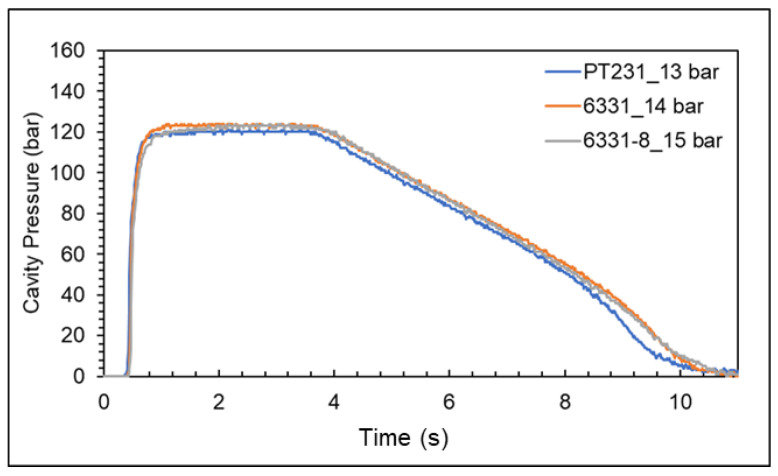
The cavity pressure profile of three materials with different viscosities at appropriate packing pressure.

According to the literature, using materials with lower viscosity needs a higher clamping force setting to prevent flashes [20]. However, in this study, the appropriate clamping force for materials with different viscosities is 16 tons. The reason is that the previous optimization experiments of the cavity pressure profile are very close, as shown in Figure 22.

Under the setting of clamping force at 16 tons, the clamping force difference value is zero for 6331-8, 6331, and PT231. To prevent the instability of the injection-molding machine and the influence of different batches of materials, the appropriate clamping forces should be multiplied by 1.2 times. Thus, the final appropriate clamping force setting is 19 tons.

## 5. Adaptive Process Control System

**Figure 23:**
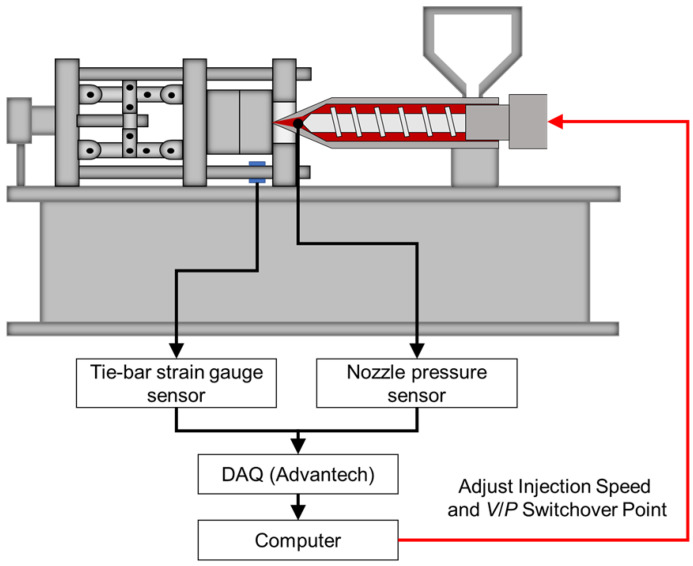
Adaptive process control system.

An adaptive process control system is a type of control system with the ability to adapt to environmental variation and adjust the parameters of the system autonomously. This research develops a control system that can revise the *V/P* switchover point and injection speed automatically based on the viscosity index, nozzle peak pressure, and clamping force difference value to stabilize the product weight, as shown in Figure 23. The main parameters of the adaptive process control system are obtained from the optimization experiments in Chapter 4. In addition, other process parameters are referred to in previous studies in the same field [16,18]. After the optimization experiments, the adaptive process control system needs to set a qualified quality range through the quality characteristics such as nozzle pressure peak or viscosity index to define whether the product quality is acceptable or not. Then, the experiments using optimized process parameters as the starting point and adaptive process control system are carried out. If the characteristics exceed the upper or lower limits, the system will adjust the corresponding parameters autonomously, such as *V/P* switchover point and injection speed, to stabilize the product quality for the next mold. Through the experiments, it is verified that the use of an adaptive process control system in production can effectively enhance the stability of quality.

### 5.1. Control Strategy of Adaptive Process Control System

The nozzle peak pressure and the viscosity index are found to be highly correlated with the product weight [17,19]. So, in the adaptive process control system of this study, the nozzle peak pressure and the viscosity index are used as the quality indexes to monitor the variation of the part weight during the injection-molding process.

Through the experiments, it is found that setting the *V/P* switchover point too late will cause excessive compression of the mold cavity, which will lead to a sharp rise in the nozzle peak pressure and flash in the product. If the *V/P* switchover point is set too early, the nozzle peak pressure will not be high enough due to insufficient material quantity, and the product will have possible shrinkage. Therefore, the nozzle peak pressure can be controlled by adjusting the *V/P* switchover point.

The viscosity index in this study is a pressure–time integral from the start of the injection stage to the end of the cooling stage. The filling time is shorter under high injection speed. Therefore, the viscosity index will decrease when the injection speed increases. Based on the *P-V-T* relationship, high injection speed will cause the melt temperature to rise and the specific volume to increase due to the shear effect, and the product weight will show a downward trend. In summary, the viscosity index and product weight are negatively correlated, so the viscosity index can be controlled by adjusting the injection speed to stabilize the product weight [20]. 

It was found that when the clamping force difference value is zero, the mold will not separate [9]. Therefore, the clamping force difference was also monitored to prevent the mold separation.

The process of automatic parameter adjustment is that when the system detects that the quality characteristics exceed the upper or lower limits, it will transmit the parameter adjustment command to the control panel of the injection-molding machine, and the machine will autonomously adjust the injection parameters of the next mold. The entire process is executed automatically by the system itself.

**Figure 24:**
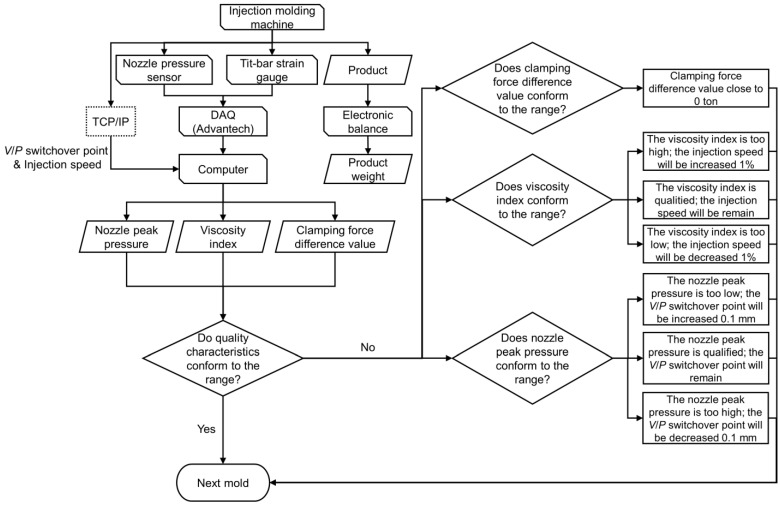
Flow chart of adaptive process control system.

The control strategy of the adaptive process control system is as follow: (1) When the nozzle peak pressure is too high, the *V/P* switchover point will be reduced 0.1 mm. When the nozzle peak pressure is too low, the *V/P* switchover point will be increased 0.1 mm by the system. (2) When the viscosity index is too high, the injection speed will be increased 1%. When the viscosity index is too low, the injection speed will be reduced 1% by the system. (3) Monitor the clamping force difference value close to zero value to make sure the mold was not separated. Figure 24 presents the flow chart of the adaptive process control system.

### 5.2. Experiment Results of the Adaptive Process Control System

In this experiment, one hundred cycles of production were used to compare the stability of the weight of product using or not using the adaptive process control system. Table 6 shows the adaptive process control system’s experiment parameters. The *V/P* switchover point, injection molding, packing pressure, and clamping force are the optimized parameters. The variation and standard deviation of the product weight are calculated to determine the product weight stability. In this research, the standard of variation of the product weight was defined as less than 0.2%, and the standard of standard deviation of the product weight was defined as less than 0.03 g.

(4) $$\mathrm{Variation}=\frac{W_{\max}{\;-\;W}_{\min}}{W_{\mathrm{average}}}\times 100\%$$

(5) $$\sigma =\sqrt{\frac{\sum_{i=1}^{N}{\left(\mathrm{xi}\;-\;\bar{x}\right)}^{2}}{N}}$$

Here, Variation is the variation of the product weight, *W_max_* is the maximum of the product weight, *W_min_* is the minimum of the product weight, *W_average_* is the average of the product weight, σ is the standard deviation of the product weight, $\bar{x}$ is the average of the product weight, and *N* is the number of the product.

**Figure 25:**
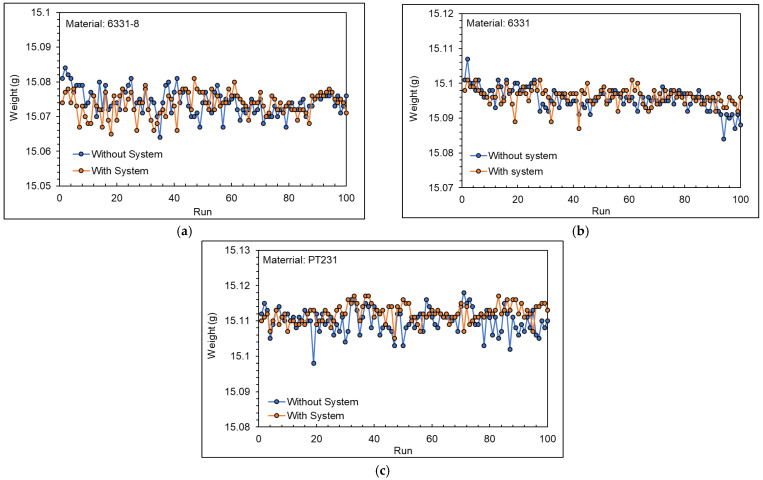
Product weight with and without the adaptive process control system experiments ((**a**) 6331−8, (**b**) 6331, and (**c**) PT231).

Figure 25 shows the product weight with and without the adaptive process control system. Table 7 shows the variation of the product weight. Table 8 shows the standard deviation of the product weight. The experiment results show that when not using the system, the variation and standard deviation of the product have met the goal of research with the optimized process parameters. When using the system, the stability of product weight has been further improved.

**Figure 26:**
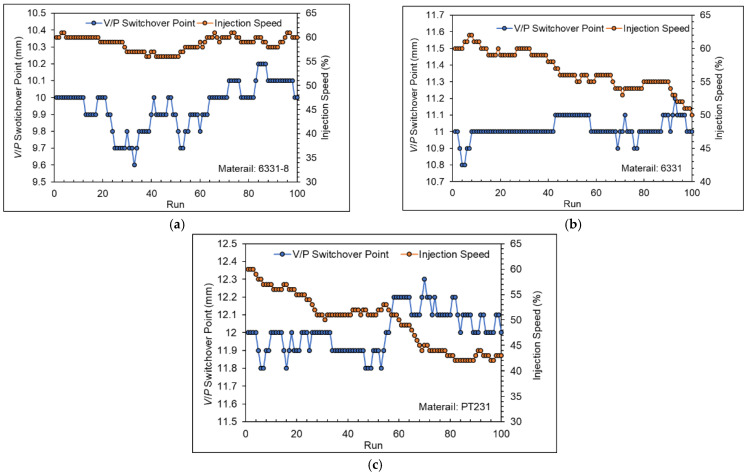
Adjustment process of the adaptive process control system ((**a**) 6331−8, (**b**) 6331, and (**c**) PT231).

Figure 26 shows the adjustment process of the adaptive process control system. The results show that the adaptive process control system will revise the *V/P* switchover point and injection speed automatically when the viscosity index and nozzle peak pressure cross the upper and lower limit.

**Figure 27:**
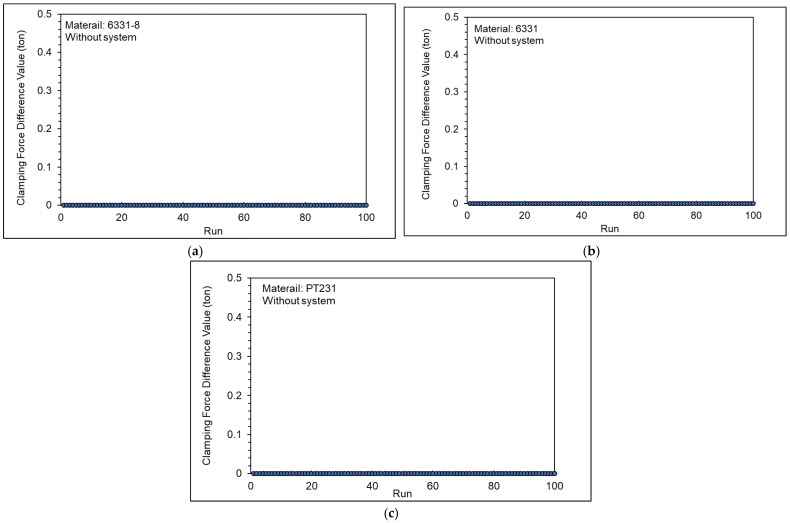
Clamping force difference value with adaptive process control system ((**a**) 6331−8, (**b**) 6331, and (**c**) PT231).

Figure 27 shows the clamping force difference value with the adaptive process control system. The results show that the mold was not separated when the adaptive process control system adjusted the *V/P* switchover point or injection speed. This means that the clamping force of 19 tons is sufficient for the adaptive process control system to adjust the injection-molding parameters, and the mold is not separated.

## 6. Conclusions

This study establishes a procedure to define standard process parameters based on the results of two sensors, which are based on nozzle peak pressure, timing of peak pressure, pressure difference, and clamping force difference values to optimize the *V/P* switchover point, injection speed, packing pressure, and clamping force. In addition, this research develops a control system that can revise the *V/P* switchover point and injection speed automatically in order to stabilize the product weight. Finally, this research validates the optimization procedure and control system by using three different viscosities’ materials. The experiment results support the following significant findings and conclusions:

1. When the cavity is completely filled, the behavior of the melt changes from the flowing stage to the compressing stage, which causes a sudden rise in nozzle pressure. The nozzle peak pressure can be used to optimize the *V/P* switchover point.
2. To avoid the melt’s over-compression and to make injection-molding cycle time as short as possible, the injection speed needs to be adjusted. The nozzle peak pressure and timing of peak pressure can be used to optimize the injection speed.
3. Packing pressure setting should consider the product quality, for example, product weight. Product weight and pressure difference can be used to optimize the packing pressure.
4. When the clamping force difference value is zero, it is the appropriate clamping force.
5. Using three materials with different viscosities under the adaptive control system, the variation of product weight was reduced to 0.106%, 0.092%, and 0.079%. In addition, the standard deviation of the product weight was reduced to 0.0034 g, 0.0025 g, and 0.0025 g.

## Figures and Tables

**Table 1:** Status of mold separation [7].

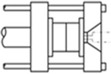	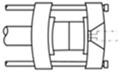	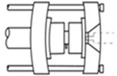
Mold compression High clamping force setting Possible venting problem	Slight mold separation	Significant mold separation Too-low clamping force setting Possible flash defect

**Table 2:** *V/P* switchover point experiment parameters.

Fixed Parameters			
**Injection pressure (bar)**	**Cooling time (s)**	**Packing time (s)**	**Mold temperature (°C)**
170	15	0.1	60
**Melting temperature (°C)**	**Injection speed (%/(mm/s))**	**Packing pressure ** **(bar)**	**Clamping force (ton)**
210	50/81.35	10	40
**Changed parameter**			
***V*/*P* switchover point (mm)**	**6331-8**	**6331**	**PT231**
	7, 8, 9, 10, 11	8, 9, 10, 11, 12	9, 10, 11, 12, 13

**Table 3:** Injection speed experiment parameters.

Fixed Parameters			
**Injection pressure (bar)**	**Cooling time (s)**	**Packing time (s)**	**Mold temperature (°C)**
170	15	0.1	60
**Melting temperature (°C)**	**Packing pressure ** **(bar)**	**Clamping force ** **(ton)**	
210	10	40	
***V/P* switchover point (mm)**	**6331-8**	**6331**	**PT231**
	10	11	12
**Changed parameter**			
**Injection speed (%/(mm/s))**	30/48.81, 40/65.08, 50/81.35, 60/97.62 70/113.89, 80/130.16, 90/146.43		

**Table 4:** Packing pressure experiment parameters.

Fixed Parameters			
**Injection pressure (bar)**	**Cooling time (s)**	**Packing time (s)**	**Mold temperature (°C)**
170	15	3	60
**Melting temperature (°C)**	**Injection speed (%/(mm/s))**	**Clamping force ** **(ton)**	
210	60/97.62	40	
***V/P* switchover point (mm)**	**6331-8**	**6331**	**PT231**
	10	11	12
**Changed parameter**			
**Packing pressure ** **(bar)**	**6331-8**	**6331**	**PT231**
	4, 5, 6, …, 23, 24, 25	4, 5, 6, …, 18, 19, 20	4, 5, 6, …, 18, 19, 20

**Table 5:** Clamping force experiment parameters.

Fixed Parameters			
**Injection pressure (bar)**	**Cooling time (s)**	**Packing time (s)**	**Mold temperature (°C)**
170	15	3	60
**Melting temperature (°C)**	**Injection speed (%/(mm/s))**		
210	60/97.62		
***V/P* switchover point (mm)**	**6331-8**	**6331**	**PT231**
	10	11	12
**Packing pressure ** **(bar)**	**6331-8**	**6331**	**PT231**
	15	14	13
**Changed parameter**			
**Clamping force ** **(ton)**	10, 11, 12, …, 18, 19, 20		

**Table 6:** Adaptive process control system’s experiment parameters.

Fixed Parameters			
**Injection pressure (bar)**	**Cooling time (s)**	**Packing time (s)**	**Mold temperature (°C)**
170	15	3	60
**Melting temperature (°C)**	**Injection speed (%/(mm/s))**		
210	60/97.62		
***V/P* switchover point (mm)**	**6331-8**	**6331**	**PT231**
	10	11	12
**Packing pressure** **(bar)**	**6331-8**	**6331**	**PT231**
	15	14	13
**Clamping force** **(ton)**	19		

**Table 7:** Variation of product weight with and without the system.

Material	Without System (%)	With System (%)
6331-8	0.112	0.106
6331	0.152	0.092
PT231	0.132	0.079

**Table 8:** Standard deviation of product weight.

Material	Without System (%)	With System (%)
6331-8	0.0037	0.0034
6331	0.0033	0.0025
PT231	0.0035	0.0025

**Figure A1:**
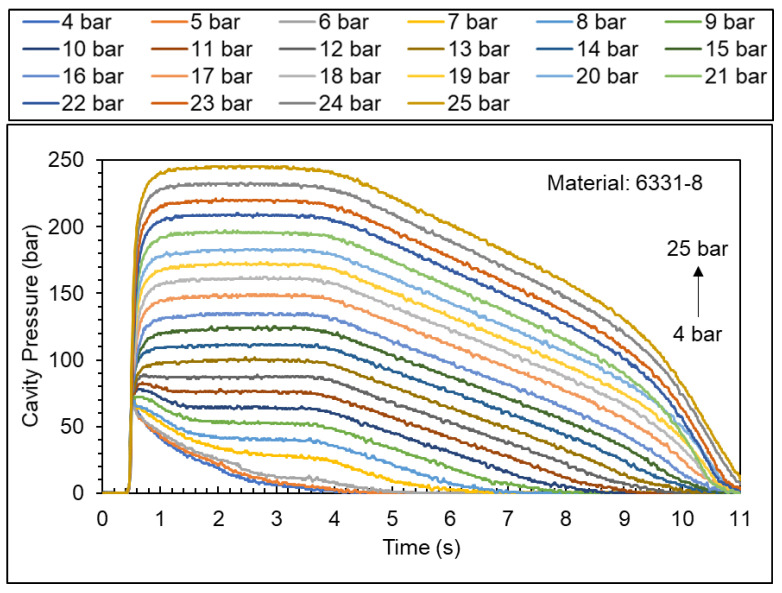
Cavity pressure profiles of 6331−8 at different packing pressures.

**Figure A2:**
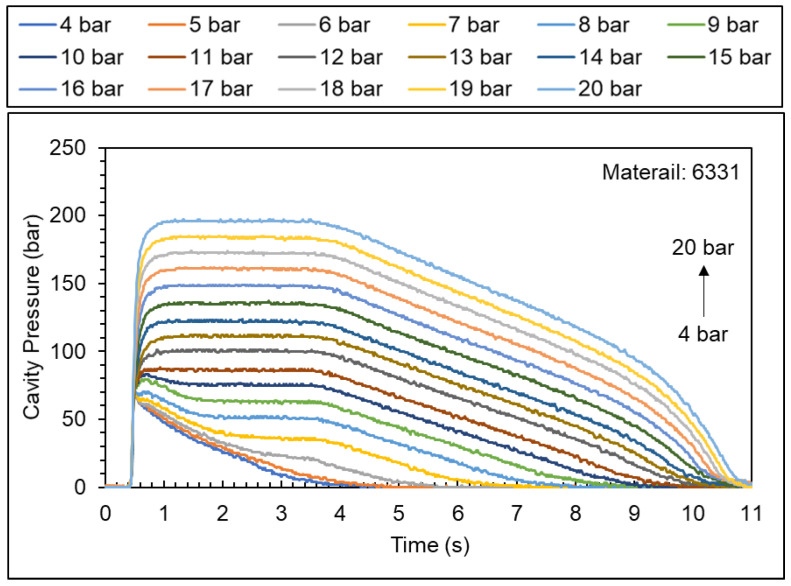
Cavity pressure profiles of 6331 at different packing pressures.

**Figure A3:**
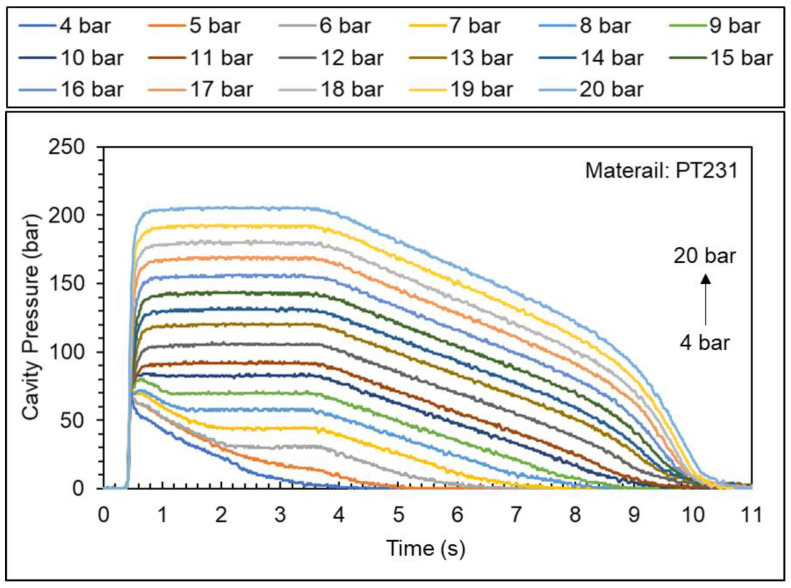
Cavity pressure profiles of PT231 at different packing pressures.

**Figure A4:**
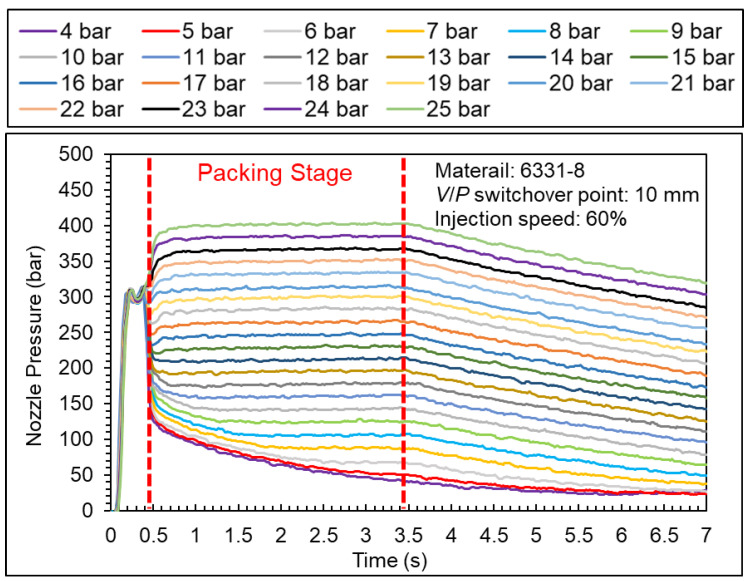
Nozzle pressure profiles of 6331−8 at different packing pressures.

**Figure A5:**
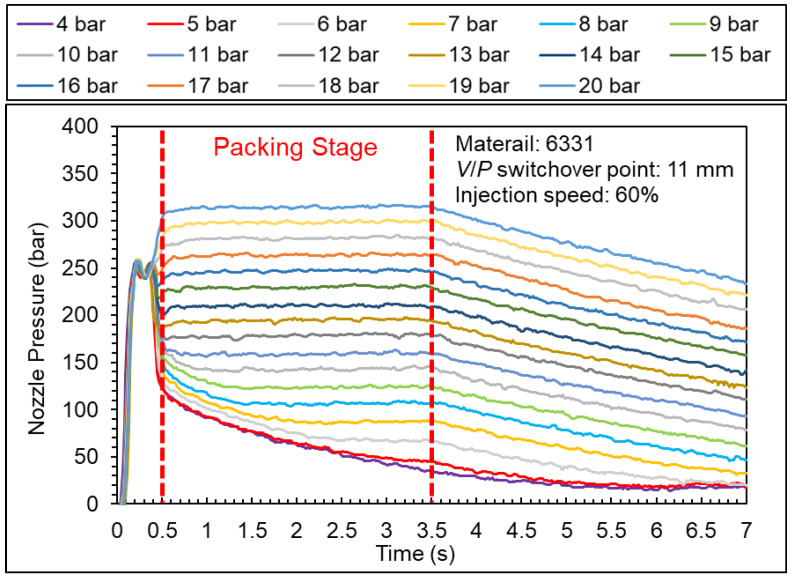
Nozzle pressure profiles of 6331 at different packing pressures.

**Figure A6:**
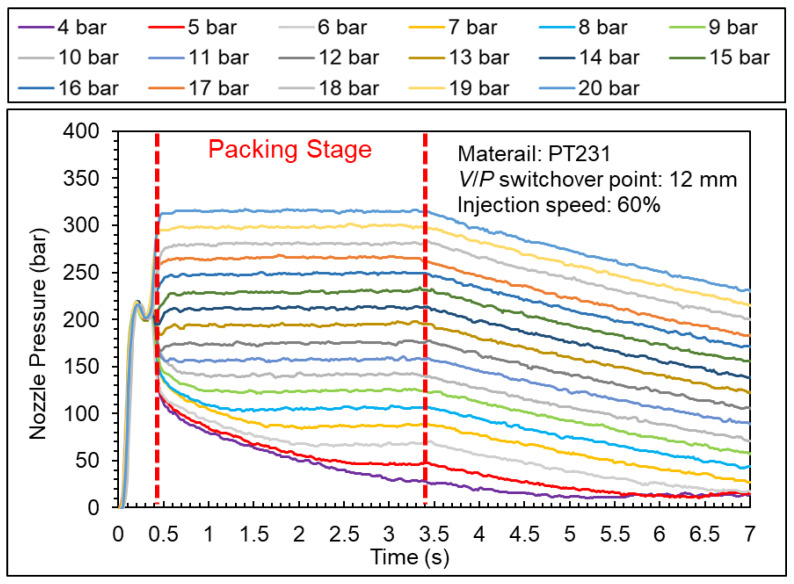
Nozzle pressure profiles of PT231 at different packing pressures.

## Data Availability

The data presented in this study are available on request from the corresponding author.
